# Supplementary material for: Treatment and Outcomes of Metastatic Non-Small-Cell Lung Cancer Harboring Uncommon EGFR Mutations: Are They Different from Those with Common EGFR Mutations?
Source: Biology (Basel). 2020 Oct 7;9(10):326. doi: 10.3390/biology9100326 (PMC7600176; doi:10.3390/biology9100326)

Supplementary Table 1

Comparison of the baseline characteristics between common and uncommon EGFR mutation NSCLC

Patient Characteristics	Total N	Common mutation	Uncommon mutation	*P*-value
Total	2,121	1,986	135	
Age				
<60 years	906 (42.7%)	859 (43.3%)	47 (34.8%)	0.06
≥60 years	1215 (57.3%)	1127 (56.7%)	88 (65.2%)	
Sex				
Male	780 (36.8%)	716 (36.1%)	64 (47.4%)	0.02
Female	1341 (63.2%)	1270 (63.9%)	71 (52.6%)	
ECOG PS				
0	442 (20.9%)	414 (20.8%)	28 (20.7%)	0.247
1	1573 (74.2%)	1477 (74.4%)	96 (71.1%)	
2	106 (5.0%)	95 (4.8%)	11 (8.1%)	
Smoking status				
Never smoker	1484 (70.0%)	1409 (70.9%)	75 (55.2%)	0.003
Ex-smoker	432 (20.4 %)	387 (19.5%)	45 (33.3%)	
Current smoker	205 (9.7%)	190 (9.6%)	15 (11.1%)	
History of curative thoracic surgery				
Yes	685 (32.3%)	659 (33.2%)	26 (19.2%)	0.55
No	1436 (67.7%)	1327 (66.8%)	109 (80.7%)	
EGFR-TKI				
Gefitinib	1046 (49.3%)	999 (50.0%)	47 (38.5%)	0.043
Erlotinib	305 (14.4%)	284 (14.2%)	21 (17.2%)	
Afatinib	770 (36.3%)	716 (35.8%)	54 (44.3%)	
Patients who experienced disease progression	1316 (62.0%)	1,238 (61.9%)	78 (63.9%)	0.70
Sequential treatment				
Rate of re-biopsy after failing first-line EGFR-TKIs	859 (65.3%, 859/1316)	824 (66.6%, 824/1238)	35 (44.9%, 35/78)	<0.001
Detection rate of T790M	533 (62.0%, 533/859)	523 (63.5%, 523/824)	10 (28.6%, 10/35)	<0.001
Sequential treatment				
Third-generation EGFR-TKIs	599(45.5%)	582 (47.0%)	17 (21.8%)	<0.001
Cytotoxic chemotherapy	488 (37.1%)	447 (36.1%)	41 (52.6%)	
No sequential treatment	229 (17.4%)	209 (16.9%)	20 (25.6%)	

Supplementary Fig. 1. (A) Median progression-free survival (PFS) according to the type of epidermal growth factor receptor *(EGFR)* mutation (common versus uncommon). (B) Median overall survival (OS) according to the type of EGFR mutation (common versus uncommon)

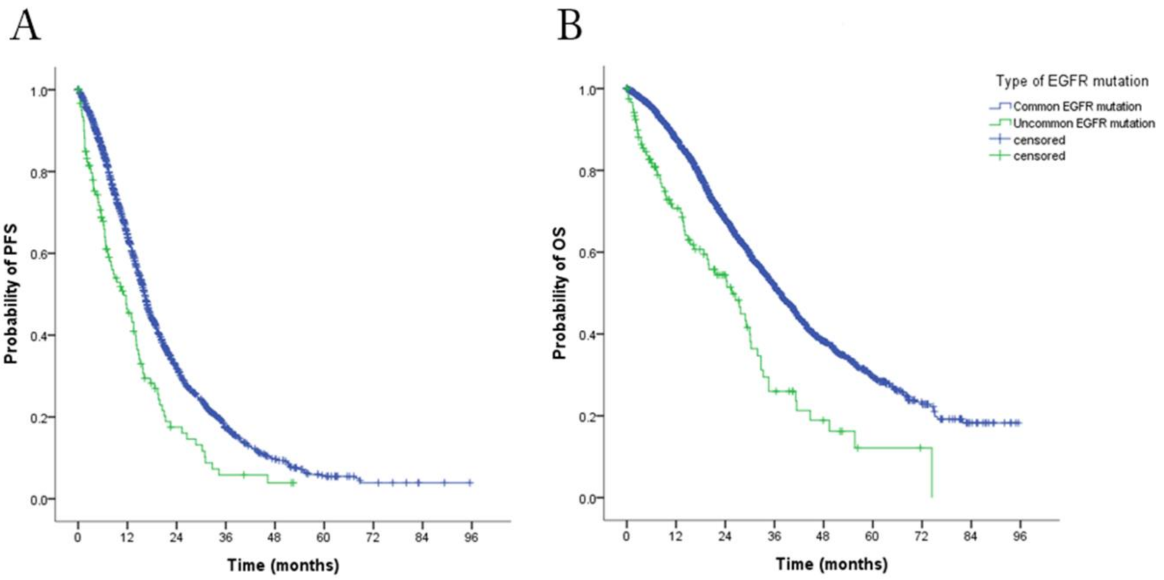

Supplement: Supplementary file 1 [file biology-09-00326-s001.pdf]
